# Corrosion Behavior of AISI 304 Stainless Steel Reinforcements in SCBA-SF Ternary Ecological Concrete Exposed to MgSO_4_

**DOI:** 10.3390/ma13102412

**Published:** 2020-05-24

**Authors:** Hilda A. Ariza-Figueroa, Juan Bosch, Miguel Angel Baltazar-Zamora, René Croche, Griselda Santiago-Hurtado, Laura Landa-Ruiz, José M. Mendoza-Rangel, José M. Bastidas, Facundo Almeraya-Calderón, David M. Bastidas

**Affiliations:** 1 Facultad de Ingeniería Mecánica y Eléctrica (FIME), Doctorado en Ingeniería, Universidad Veracruzana, Xalapa 91000, Veracruz, Mexico; hilda_af@hotmail.com; 2 Department of Chemical, Biomolecular, and Corrosion Engineering, National Center for Education and Research on Corrosion and Materials Performance (NCERCAMP-UA), The University of Akron, 302 E Buchtel Ave, Akron, OH 44325-3906, USA; jb394@zips.uakron.edu; 3 Facultad de Ingeniería Civil-Xalapa, Universidad Veracruzana, Lomas del Estadio S/N, Zona Universitaria, Xalapa 91000, Veracruz, Mexico; lalanda@uv.mx; 4 Facultad de Ingeniería Mecánica y Eléctrica, Universidad Veracruzana, Xalapa 91000, Veracruz, Mexico; rcroche@uv.mx; 5 Facultad de Ingeniería Civil—Unidad Torreón, Universidad Autónoma de Coahuila, Torreón 27276, Mexico; grey.shg@gmail.com; 6 Facultad de Ingeniería Civil, Universidad Autónoma de Nuevo León, Ave. Pedro de Alba S/N, Ciudad Universitaria, San Nicolás de los Garza 66455, Mexico; jmmr.rangel@gmail.com; 7 National Centre for Metallurgical Research (CENIM), CSIC, Ave. Gregorio del Amo 8, 28040 Madrid, Spain; bastidas@cenim.csic.es; 8 Universidad Autónoma de Nuevo León, FIME—CIIIA, Av. Universidad S/N, Ciudad Universitaria, San Nicolás de los Garza 66455, Mexico

**Keywords:** corrosion, ternary ecological eoncrete, sugar cane bagasse ash, silica fume, AISI 304, sulfates

## Abstract

In this study, ternary ecological concrete (TEC) mixtures were produced with partial substitution of the ordinary Portland cement (OPC) by 10%, 20%, and 30% of sugar cane bagasse ash (SCBA) and silica fume (SF); a control mixture (100% OPC) was prepared according to ACI 211.1 standard. The studied TEC specimens were reinforced with AISI 304 stainless steel and AISI 1018 carbon steel rebars. TEC reinforced specimens were immersed in two different electrolytes, a control (DI-water) and 3.5 wt.% MgSO_4_ solution, for 180 days. The electrochemical corrosion was monitored by corrosion potential (*E_corr_*) according to ASTM C-876-15 standard, and the linear polarization resistance (LPR) technique using ASTM G59 standard. The *E_corr_* and current density *i_corr_* results show that AISI 304 stainless steel rebars have a high corrosion resistance, with *i_corr_* values below 0.1 µA/cm^2^, which is interpreted as a level of negligible corrosion. The best corrosion performance was found for the TEC mixture made with a 20% addition of blend of sugar cane bagasse ash-silica fume (SCBA-SF) to the OPC.

## 1. Introduction

Concrete is the most widely used building material worldwide, owing to its excellent physical properties including excellent mechanical properties and durability. Reinforced concrete is of paramount importance for the development of societies, which demand advanced civil engineering structures and infrastructure, such as bridges, buildings, pavements, dams, pipelines, and canisters, among others. However, the corrosion of reinforcing steel in concrete is the main cause of premature deterioration of the infrastructure and one of the most important issues for the maintenance of the structural integrity, which dramatically impacts economy [1,2,3,4,5,6]. The corrosion of steel embedded in concrete is an electrochemical process influenced by the chloride ingress and carbonation [7].

The use of stainless steel (SS) reinforcement is an efficient method for preventing the corrosion of reinforced concrete (RC) structures [8,9]. Ferritic and austenitic stainless steels were the first SS reinforcements developed; currently, the tendency is the use of duplex SS (DSS). The SS passivates in the atmosphere, but when in contact with the alkaline environment of the concrete, this passive layer is not stable and a new passivation process takes place [10,11,12]. Several studies with stainless steels reported their good corrosion resistance in chloride polluted environments, which vary in function of the SS chemical composition, the type of test performed (accelerated, natural), and the media (pore solution, mortar, or concrete) [13,14,15,16,17,18,19].

The corrosion process can be caused by several factors, the most significant of which is the entry of the aggressive ions, such as chlorides present in marine environments [20,21,22] and sulfates, which are inorganic salts normally present in the ground [23,24,25,26], as well as in groundwater and in surface water, although the degree of concentration can be highly variable. The presence of sulfates in water in contact with a hardened cement paste can significantly increase the solubility of components of the concrete admixture and cause degradation of concrete through leaching; therefore, the steel remains unprotected [27,28,29]. This concrete degradation can lead to a severe structural failure as the reinforcement material is more susceptible to corrosion processes. Through the use of non-destructive tests (NDTs) and/or analytical formulation, which are fast and high quality methods to assess the corrosion of reinforcing steel, according to the determination of the lost cross section of the bar, using novel analytical models [30], the section loss due to corrosion products at the steel/concrete interface of specimens subjected to different environments has originated a generalized concept of paste filled with corrosion product (CP) [31]. A widely used strategy to mitigate this reinforcement corrosion is to use coatings in order to substantially increase the durability of the structure. A similar effect can be generated using additives to the concrete such as sealants that improve the corrosion protection of the reinforcement. Moreover, laboratory simulations show that the concrete reinforced with galvanized steel is better in an aggressive environment, as well as resisting contaminants found present in the concrete mixture itself [32,33,34].

The production of ordinary Portland cement (OPC) generates between 5% and 8% CO_2_ total emissions to the environment and could increase to between 10% and 15% in the future [35]. Therefore, different approaches and solutions to retard or reduce the corrosion process and mitigate emissions produced from the cement industry have been investigated. The solutions proposed to reduce these high emissions include new alkali-activated materials, such as fly ash (FA), slags, or metakaolin, among others [36]. Furthermore, in the last 20 years, sugar cane bagasse ash (SCBA) and rice husks ash (RHA) have been studied in order to provide a more sustainable and equally performing solution for reinforced structures [37,38] coming from agricultural waste, as less FA resources were available owing to the coal regulations [39]. SCBA is a sugar mill byproduct obtained from the bagasse combustion that, after being treated, can be used a concrete binder thanks to its pozzolanic activity. However, few corrosion studies have been performed for all these novel reinforced concretes. For that reason, the most conservative approach, because of the lack of agreement about their corrosion performance, is to use them as supplementary cementitious materials (SCMs), gradually replacing the OPC with low quantities of the novel materials. This replacement is an environmentally friendly, and cost-effective solution owing to the by-products nature of these novel materials [40,41]. Another important factor of these novel materials is that they cure faster than OPC [42], making them even more suitable for precast components.

These SCMs promote a reaction known as the pozzolanic reaction, wherein the Ca(OH)_2_ from the hydration process reacts with these additives, decreasing the porosity and permeability of the concrete [43]. The benefits were mainly derived from the presence of a high SiO_2_ content and amorphous mineralogical character in the SCMs, allowing the calcium hydroxide (free lime forms during cement hydration) to react with the silica content present in the pozzolanic materials and water, which forms additional calcium silicate hydrate; this is called secondary gel, hence the compressive strength is enhanced [44]. Ultra-fine particles of silica fume enhance the concrete in the hardened state by acting as a micro filling material in the concrete, which fills the micro-voids in concrete. Densification of the interfacial transition zone also takes place, as well as further enhancing of the matrix aggregate bond—the enhancement in strength due to the additional formation of C–S–H gel resulting from the pozzolanic reaction between silica fume and calcium hydroxide [45]. Several SCMs have been reported to have different behaviors regarding chloride ingress, carbonation, and sulfates resistance. Even between the same materials, different behaviors were reported, leading to a lack of agreement. For instance, FA reported a higher chloride ingress resistance than OPC [46,47], but a similar chloride penetration was also reported by Ganesa et al. [48]. By adding metakaolin to the FA, not only is the geopolymerisation process enhanced, but also the porosity is decreased, hence improving the chloride ingress resistance [49,50]. However, the slags contribute to improved carbonation and chloride ingress resistance, as stated by Navarro et al. [51]. Silica fume (SF) addition to OPC showed decreased chloride threshold values [52], but an increased chloride resistance performance was shown in different studies at the same time [53,54]. It also increases the freeze–thaw resistance, as stated by Gruszczyński et al. [55]. Although SCBA has workability issues, an addition between 10% and 30% as an OPC replacement to the mixture reduces not only the permeability, but also the diffusion of chloride ions through the concrete [56,57,58,59,60,61]. However, the post-treatment can also affect these results by increasing the greenhouse emissions or by decreasing the workability of these concretes, apart from the mechanical and chemical properties as stated by Franco-Luján et al. [61]. As can be seen from the literature review, there is an existing lack of agreement between authors about the corrosion performance of these novel materials. This lack of agreement might be because of the manufacturing processes, mixture design, curing conditions, or exposure conditions. For that reason, further development has to be done in order to determine the mechanisms behind this corrosion performance behavior and to generate a solution to the high pollutant OPC.

The aim of this work was to study the corrosion behavior of partially substituted SCBA and SF ternary ecological concrete (TEC) mixtures embedding AISI 304 SS and AISI 1018 carbon steel (CS) rebars. TEC has been used as an alternative material to OPC in this work owing to its pozzolanic characteristics [62], as partial substitutes of the OPC, to generate a reduction in CO_2_ and achieve improvements in the concrete properties to improve their performance against corrosion of the reinforcing steel, as has been proposed in some recent projects [63,64]; few proposals have used it for soil improvement [65]. Four concrete mixtures were produced according to the ACI 211.1 standar [66], the first with 100% OPC, while the remaining three were prepared with substitutions partially in percentages of 10%, 20%, and 30% of the OPC in combination with SCBA and SF TEC.

## 2. Materials and Methods

### 2.1. Ternary Ecological Concrete

In this investigation, OPC was used, in accordance with the NMX C-414 standard of the ONNCCE [67]. SCBA and SF were used as partial substitutes for OPC with replacement percentages of 10%, 20%, and 30%, thus producing TEC mixtures. The SCBA was obtained from Sugar Mills Mahuixtlan, located in Coatepec, Mexico. The SCBA was sampled from one of the boilers where the combustion temperature reached 750 °C. The SF used was purchased from a commercial supplier.

The physical characterization of the aggregates was performed, according to the following tests: ASTM C33/C33M–16e1 (Standard Specification for Concrete Aggregates) to determine the fineness modulus and maximum aggregate size [68], ASTM C29/C29M–07 (Standard Test Method for Bulk Density (Unit Weight) and Voids in Aggregate) [69], ASTM standards: ASTM C-127-15 (Standard Test Method for Relative Density (Specific Gravity) and Absorption of Coarse Aggregate) [70], and ASTM C-128-15 (Standard Test Method for Relative Density (Specific Gravity) and Absorption of Fine Aggregate) [71]. All the results obtained from the physical characterization of the aggregates are summarized in Table 1.

### 2.2. Proportioning of Concrete Mixtures

Concrete mixtures were designed in accordance to ACI 211.1 method [66], the most used design method for concrete research [72,73,74,75]. This method is based on the physical properties for coarse and fine aggregates, see Table 1.

Four different hydraulic concrete mixtures were prepared, with the control mixture with 100% OPC and three mixtures with partial substitution of 10%, 20%, and 30% of the OPC with combinations of SCBA-SF. Table 2 the shows the dosage used for each concrete mixture, considering the four concrete mixes an F´c = 29.4 MPa (compressive strength).

### 2.3. Characterization of Fresh and Hardened Concrete

The characterization of fresh concrete was performed using slump testing, temperature measurements, and volumetric mass (density) measurements performed according to ONNCCE and ASTM standards; the results obtained for each mixture can be seen in Table 3.

Table 4 presents the results of the compressive strength (concrete in the cured state) of the four mixtures studied, and assays were performed after 7, 14, and 28 days, as indicated by the standard NMX-C-083-ONNCCE-2002 [79].

### 2.4. Characteristic and Nomenclature of Test Specimens

The control OPC mixture and the three mixtures of TEC were made a with water-to-cement ratio of 0.65. The specimens were prisms of with dimensions of 15.0 × 15.0 × 15.0 cm. In all the specimens, AISI 304 and AISI 1018 steel bars were embedded. The steel bars had a length of 15 cm and 9.5 mm diameter. The curing of all specimens was carried out by immersion in DI-water for 27 days, according to NMX-C-159 standard [80]. After the curing period, the eight specimens were placed in the exposure media, four specimens were immersed in DI-water (control medium), and the remaining four were immersed in 3.5 wt.% MgSO_4_ solution for 175 days, simulating a sulfated medium or aggressive medium. The specimens were then subjected to electrochemical tests. Table 5 shows the elemental composition analyzed by X-ray fluorescence spectroscopy (XRF) of the AISI 304 austenitic stainless steel (SS) and AISI 1018 carbon steel (CS).

The nomenclature used for the electrochemical monitoring of corrosion potential and corrosion kinetics of AISI 304 SS and AISI 1018 CS embedded in TEC, exposed to DI-water (control medium) and 3.5 wt.% MgSO_4_ solution (aggressive medium), is shown in Table 6.

**Figure 1:**
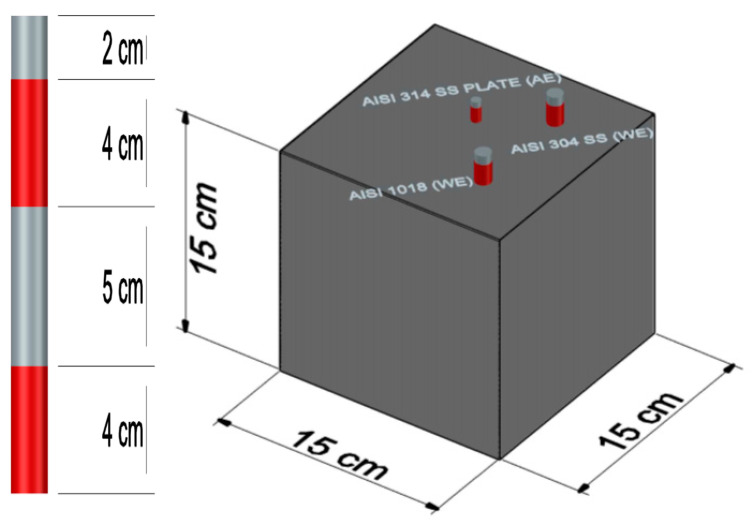
Experimental design of the rebars and concrete specimens.

Prismatic-reinforced TEC specimens were manufactured using AISI 1018 CS and AISI 304 SS reinforcements, with a 15 cm length and 9.5 mm diameter, as depicted in Figure 1. Each of the bars were coated 4 cm from the top and 4 cm from the bottom, in order to delimit the area of exposure to corrosion of steel in concrete with a length of 5 cm.

TEC specimens were exposed to two different electrolytes, control medium (DI-water) and 3.5 wt.% MgSO_4_ solution, for a period of 182 days.

Electrochemical measurements were performed using a conventional three-electrode cell configuration. The AISI 1018 CS and AISI 304 SS were used as the working electrodes (WEs). A standard copper/copper sulfate (Cu/CuSO_4_) and a AISI 314 SS plate were used as reference (RE) and counter electrode (AE), respectively. The half-cell corrosion potential (*E_corr_*) according to ASTM C876-15 standard [81] and considering one more range, according to the literature [82]. The linear polarization resistance (LPR) was recorded at a sweep rate of 10 mV/min at, a potential scan range was applied between −20 to +20 mV versus (Cu/CuSO_4_), according to ASTM G59-97 standard [83]. Electrochemical measurements were performed in a Gill AC Galvanostat/Potentiostat/ZRA (ACM Instruments, Cark in Cartmel, UK), the results were analyzed using Version 4 Analysis specialized software from ACM Instruments (Cark in Cartmel, UK) [84,85].

The TEC reinforced specimens were immersed in the 3.5 wt.% MgSO_4_ solution at room temperature, and *E_corr_* and *i_corr_* were monitored every two weeks and all experimental measurements were carried out in triplicate.

The *i_corr_* and the corrosion rate (*v_corr_*) were estimated from the LPR technique using Stern and Geary Equation (1) [86]:

(1) $$i_{corr}=\frac{B}{Rp}$$

where *Rp* is expressed in Ω∙cm^2^ and *B* in V is a constant resulting from a combination of the anodic and cathodic Tafel slopes; B is a constant with a recommended value of 0.026 V for active and 0.052 V for the passive corrosion of steel in concrete [87,88].

*E_corr_* was used to assess the corrosion condition of reinforced concrete specimens according to ASTM C-876-15 [81], which establishes the criteria or ranges that relate the *E_corr_* values with the risk corrosion for embedded steel specimens made with OPC concrete and TEC, see Table 7 [81,82].

To determine *v_corr_* of steels embedded in the mixtures of conventional concrete and ternary ecological concrete, the *i_corr_* values were used. The criteria used to analyze the *i_corr_* results are based on the state of corrosion of steel in concrete reported in the literature [87], as shown in Table 8.

## 3. Results and Discussion

### 3.1. Corrosion Potential

The *E_corr_* of the specimens were monitored in accordance with ASTM C876-15 [81], and interpreted by the criteria presented in Table 7 [82].

#### 3.1.1. Behavior *E_corr_* Specimens in Control Medium (DI-Water)

**Figure 2:**
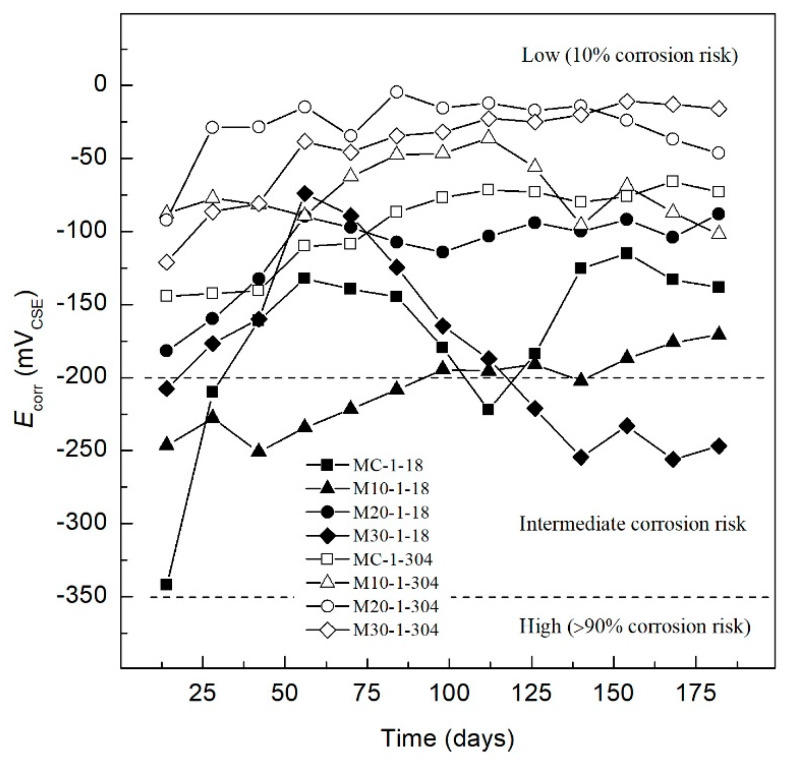
*E_corr_* of specimens exposed to control medium (DI-water).

Figure 2 shows the corrosion potential of the AISI 1018 CS and AISI 304 SS, which are embedded in different TEC, exposed in control medium (DI-water). The MC-1-18 specimen presented *E_corr_* values of −342 mV in the first 14 days, within the intermediate corrosion risk range. Subsequently, the *E_corr_* values increase to an area of low (10% of risk corrosion) according to ASTM C-876-15, with *E_corr_* values of −132 mV at day 56. Moreover, the M10-1-18 specimen presents an *E_corr_* of −227 mV in the first 28 days, and decreases after day 42, reaching a corrosion potential of −250 mV. Afterwards, *E_corr_* remains within the area of Intermediate corrosion risk until day 90, and then increases to the area with low (10% of risk corrosion) until day 180.

Meanwhile, the behavior of M20-1-18 and M30-1-18 specimens maintains a similar behavior for the first 56 days, having *E_corr_* values in the zone of low (10% of risk corrosion), showing potentials of −107 mV and −105 mV. However, the M20-1-18 specimen maintains a passive behavior with more positive values of *E_corr_* until reaching −82 mV on day 180, which indicates a low (10% of risk corrosion). The M30-1-18 specimen from day 90 to day 180 exhibits a decrease in *E_corr_* to the intermediate corrosion risk with a value of −246 mV.

For AISI 304 SS specimens, MC-1-304 shows initial values of −144 mV in the region of low (10% of risk corrosion), maintaining an electropositive growth until the end of monitoring the same area, to −73 mV in 180 days. The M10-1-304, M20-1-304, and M30-1-304 specimens maintain initial and final behavior in the region of low (10% of risk corrosion), performing better protection of TEC with substitutions of 20% and 30% of OPC by SCBA-SF combinations.

#### 3.1.2. *E_corr_* Behavior of 3.5 wt.% MgSO_4_ Solution

**Figure 3:**
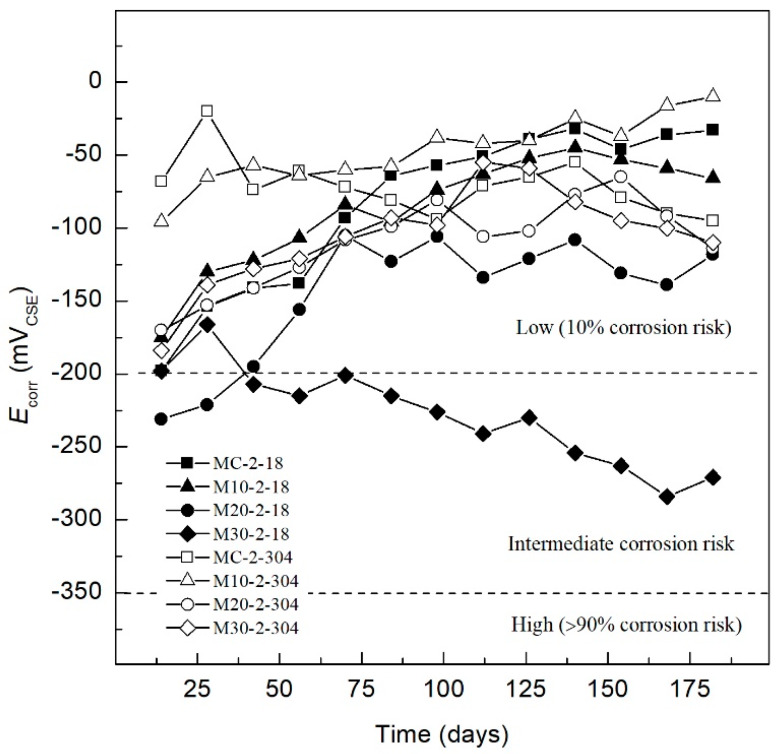
*E_corr_* of specimens exposed in 3.5 wt.% MgSO_4_ solution.

In Figure 3, in the AISI 1018 steel and 304 SS, the risk corrosion increases after being exposed to magnesium sulfate. The MC-2-18 and M10-2-18 specimens exhibit stable behavior within the area of low (10% of risk corrosion) with potentials of −198 mV and −175 mV, respectively. Finally, day 180 shows *E_corr_* values of −33 mV and −66 mV for MC-2-18 and M10-2-18 specimens, respectively. Because these specimens are more electropositive, it is apparent that the mixtures provide better protection to the steel reinforcement.

This behavior has been reported in the literature, and is associated with the reaction of sulfates with hydration products, which causes the matrix of the concrete to be denser, thus reducing the network of pores and enhancing the behavior against corrosion when exposed to sulfated media [89]. Dehwah et al. reported that the presence of sulphates in chloride solution did not affect the corrosion initiation time [90]. Meanwhile, the M20-2-18 has an initial *E_corr_* of −231 mV, within the area of uncertainty until day 28, when the specimen presents a trend towards more positive *E_corr_* values throughout the time of exposure, remaining in a range of −120 mV to −160 mV indicating a low (10% of risk corrosion), according to ASTM C-876-15. The initial *E_corr_* of sample M30-2-18 exhibits a low (10% of risk corrosion) and, after day 42, the *E_corr_* decreases until day 180, with a value −271 mV in the intermediate corrosion risk range. The MC-2-304 specimen initially presents an *E_corr_* value of −68 mV, within the area of low (10% of risk corrosion), maintaining a constant behavior within the zone. Meanwhile, MC-2-304, M10-2-304, M20-2-304, and M30-2-304 showed initial *E_corr_* values less than −200 mV, continuing with more electropositive values until day 180. The AISI 304 SS exhibits better corrosion behavior in each of the tested TEC mixtures, presenting during the 180 days of monitoring, *E_corr_* values that indicate low (10% of risk corrosion) according to ASTM C-876-15. Among the TEC specimens, M10-2-304 showed the best corrosion behaviour.

### 3.2. Corrosion Current Density

The results of the *i_corr_* values of the AISI 304 SS and AISI 1018 CS embedded in the TEC were interpreted according to the criterion of the Table 8.

#### 3.2.1. Behavior of *i_corr_* Specimens in Electrolyte: Control Medium (DI-Water)

**Figure 4:**
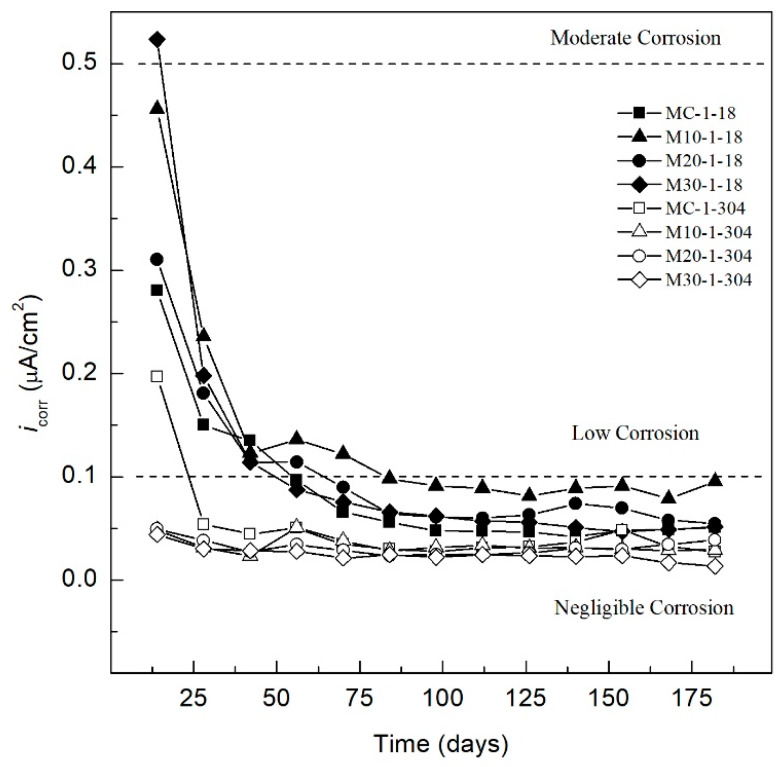
*i_corr_* specimens exposed to control medium (DI-water).

Figure 4 shows the behavior of *i_corr_* of the specimens exposed to control medium (DI-water). The specimens reinforced with AISI 1018 steel, MC-1-18, M-10-1-18, M20-1-18, and M30-1-18 show high *i_corr_* values, ranging from 0.53 to 0.28 µA/cm^2^, thus indicating a moderate corrosion according to criterion of the Table 8. The *i_corr_* values related to the formation of the passive layer that occurs in this stage of concrete mixtures (28 days) display a steady decline in values for all specimens. By day 84 of exposure to the control medium, the *i_corr_* values of the specimens were less than 0.1 µA/cm^2^, indicating a negligible level of corrosion or a passivation state of the system. A small influence is identified between the concrete types, OPC and TEC, but owing to values below 0.1 µA/cm^2^, both are considered passive.

Furthermore, the MC-1-304, M10-1-304, M20-2-304, and M30-1-304 specimens exhibit an initial *i_corr_* less than 1.0 µA/cm^2^, which indicates a negligible level of corrosion from day 14 to day 180. The *i_corr_* for specimens with AISI 304 SS coincide with values reported in the literature [91,92], monitoring below 0.03 µA/cm^2^, as well as for AISI 1018 CS when concrete is exposed in non-aggressive environments [93].

#### 3.2.2. Behavior of Specimens *i_corr_* in Electrolyte: (3.5 wt.% MgSO_4_ Solution)

**Figure 5:**
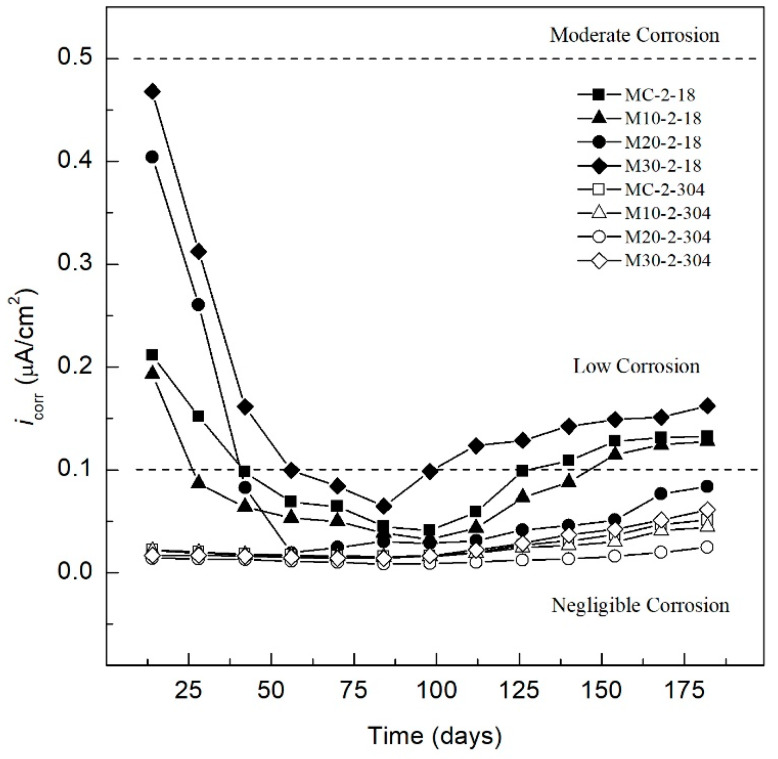
*i_corr_* specimens exposed to 3.5 wt.% MgSO_4_ solution.

Figure 5 presents the *i_corr_* of AISI 304 SS and AISI 1018 CS embedded in the TEC after 180 days of exposure to the aggressive medium (3.5 wt.% MgSO_4_ solution). The MC-2-18, M10-2-18, M20-2-18, and M30-2-18 specimens present passivity during the curing stage (up to 28 days), according to Table 6, with *i_corr_* values ranging from 0.48 to 0.08 µA/cm^2^. With increasing time, a trend towards values closer to 0.1 µA/cm^2^ can be observed, associated with passivation of the system, in agreement with the literature of concrete reinforced with AISI 1018 CS exposed to sulfates [94]. However, after 84 days of exposure, an increase in the *i_corr_* was observed in the M30-2-18 specimen, reaching values greater than 0.1 µA/cm^2^ for day 112 and 0.167 µA/cm^2^ for day 180, indicating a low level of corrosion and the activation of the M30-2-18 specimen. For the MC-2-18, M10-2-18, and M20-2-18 specimens, an increase in *i_corr_* values was observed for day 112 of exposure to the sulphates medium, associated with the activation of the steel-concrete system of the three specimens. However, the MC-2-18 specimen presents similar values to those of the M30-2-18 specimen, which exhibits an *i_corr_* value of 0.135 µA/cm^2^ at 180 days, thus displaying a better performance against corrosion than the specimen with OPC, MC-2-1. The MC-2-18 specimen shows an *i_corr_* passing the 0.1 µA/cm^2^ threshold after 140 days of exposure, ending with an *i_corr_* value of 0.127 µA/cm^2^ at 180 days of exposure, indicating the system activation and a low level of corrosion. Lastly, the specimen reinforced with AISI 1018 carbon steel, which presented the greatest resistance to corrosion when exposed to a sulphates medium, was M20-2-18, which started with an increase in its corrosion rate on day 112. Despite the increase, the specimen exhibited an *i_corr_* value of 0.0875 µA/cm^2^ by 180 days of monitoring, indicating a negligible level of corrosion. This is associated with a denser matrix and with sulfate resistance properties by replacing OPC with 10% of SCBA and 10% SF, this percentage has been reported as optimal for the improvement of the durability of ecological concrete, partially because of the pozzolanic reaction and partially to high specific surface area and the presence of reactive silica in the combination SCBA-SF [95].

For the specimens reinforced with AISI 304 SS, MC-2-304, M10-2-304, M20-2-304, and M30-2-304, exposed in magnesium sulfate medium (3.5 wt.% MgSO_4_ solution), there is a homogeneous behavior in the four specimens, with *i_corr_* values less than 0.025 µA/cm^2^ from the start of the monitoring, which indicates a negligible level of corrosion according to Table 6. The four specimens, MC-2-304, M10-2-304, M20-2-304, and M30-2-304, display *i_corr_* values in the range of 0.022 to 0.011 µA/cm^2^ in the first 28 days (curing stage). This decrease after 98 days to *i_corr_* values in a range of 0.016 to 0.009 µA/cm^2^, owing to the formation of a denser concrete matrix caused by the formation of ettringite that fills the pore network, which some authors have identified as a greater resistance to compression [96,97]. However, after day 112, a trend is identified in the four specimens. The OPC specimen, MC-2-304, and those made with TEC, M10-2-304, M20-2-304, and M30-2-304, show an increase in *i_corr_* values until the end of the monitoring (180 days). In particular, the AISI 304 steel reinforced specimen that presented the greatest protection against magnesium sulfate corrosion with the lowest *i_corr_* values was the M20-2-304 specimen, which presented *i_corr_* values that varied from 0.01 µA/cm^2^ on day 112 to 0.024 µA/cm^2^ at the end of the monitoring. Next, the specimen M10-2-304, which increased from 0.016 µA/cm^2^ on day 98 to 0.045 µA/cm^2^ on day 182. The MC-2-304 and M30-2-304 specimens show very similar *i_corr_* values, and also presented the greatest increases in *i_corr_* after day 98 of exposure, with *i_corr_* values of 0.016 µA/cm^2^ and 0.017 µA/cm^2^ for MC-2-304 and M30-2-304, respectively. By 180 days of monitoring, the *i_corr_* values increased to 0.051 µA/cm^2^ and 0.061 µA/cm^2^ for MC-2-304 and M30-2-304, respectively.

The benefit of using pozzolanic materials for protection against sulfate corrosion in reinforced concrete can be identified, with 304 SS displaying a greater protection in TEC with replacement of 20% of OPC with the combination of SCBA-SF, followed by concrete with 10% substitution. However, it should be noted that the greatest resistance to sulfate corrosion to the specimens is provided by the use of AISI 304 SS, with all specimens having values less than 0.1 µA/cm^2^, which indicates a negligible level of corrosion, owing to the high resistance that this steel presents when used as reinforcement in concrete, as has been shown by various investigations [98,99,100,101]. The sustainability of construction based on alternative materials to cement can be increased to produce concrete durable and resistant to corrosion by incorporating alternate reinforcements to common AISI 1018 CS has also been demonstrated by the use of galvanized steel [102].

## 4. Conclusions

The AISI 304 SS, when exposed to 3.5 wt.% MgSO_4_, shows excellent corrosion performance with *E_corr_* values less than −200 mV throughout the exposure period, indicating a 10% of probability of corrosion.

The corrosion rate and *i_corr_* for all specimens, including OPC and TEC, show *i_corr_* values below 0.1 µA/cm^2^, thus indicating that, after 180 days of exposure to solution at 3.5 wt.% of MgSO_4_, the level of corrosion is negligible. AISI 304 SS presented better corrosion performance in TEC (M20-2-304) with an *i_corr_* value of 0.024 µA/cm^2^ at the end of the monitoring.

These results demonstrate that the development of TEC reinforced with AISI 304 SS increases the corrosion resistance when exposed to sulfated media compared with OPC and reinforced with carbon steel AISI 1018 CS. This would significantly contribute to creating a more sustainable concrete industry.

## Figures and Tables

**Table 1:** Results of the characterization of the aggregates, obtained according to ASTM standards.

Physical Properties of Materials	Standard	Aggregate	
		Coarse	Fine
Maximum Aggregate Size (mm)	ASTM C33/C33M–16e1	19.05	-
Bulk Density (Unit Weight) (kg/m^3^)	ASTM C29/C29M–07	1433	1695
Relative Density (Specific Gravity)	ASTM C-127-15 ASTM C-128-15	2.6	2.2
Absorption (%)	ASTM C-127-15ASTM C-128-15	1.7	1.8
Fineness Modulus	ASTM C33/C33M–16e1	-	2.94

**Table 2:** Proportioning of concrete mixtures in kg for 1 m^3^ of concrete (F´c = 29.4 MPa). OPC, ordinary Portland cement; SBCA, sugar cane bagasse ash; FA, fly ash.

Materials	100% OPC	10% SCBA-SF	20% SCBA-SF	30% SCBA-SF
Cement	315	283.50	252.00	220.50
Water	205	205	205	205
SCBA	0	15.75	31.50	47.25
SF	0	15.75	31.50	47.25
Coarse aggregate	886	886	886	886
Fine aggregate	770	770	770	770

**Table 3:** Physical properties of individual employees.

Test	100% OPC 30R	10% SCBA-SF	20% SCBA-SF	30% SCBA-SF
Slump, cm [76]	7.0	6.0	5.5	5.0
Temperature, °C [77]	24.0	23.5	23.5	22.5
Density, kg/m^3^ [78]	2345.83	2307.29	2301.04	2276.04

**Table 4:** Compressive strength at 7, 14, and 28 days (F’c in MPa).

Concrete Mixture	Compressive Strength (MPa)		
	7 Days	14 Days	28 Days
MC = 100% OPC	24.3	28.3	31.2
M10 = 10% (SCBA-SF)	21.5	25.5	28.6
M20 = 20% (SCBA-SF)	22.4	26.1	30.0
M30 = 30% (SCBA-SF)	16.7	21.1	24.1

**Table 5:** Elemental composition (wt.%) by X-ray fluorescence spectroscopy (XRF) analysis of the reinforcements tested, AISI 1018 carbon steel (CS) and AISI 304 stainless steel (SS).

Steel	Element, wt.%									
	C	Si	Mn	P	S	Cr	Ni	Mo	Cu	Fe
AISI 1018 CS	0.20	0.22	0.72	0.021	0.020	0.13	0.06	0.02	0.18	Balance
AISI 304 SS	0.04	0.32	1.75	0.032	0.001	18.20	8.13	0.22	0.21	Balance

**Table 6:** Nomenclature of specimens tested for a period of 180 days.

Sample/Concrete Composition	Electrolytes			
	DI-Water		Solution 3.5 wt.% MgSO_4_	
	AISI 1018	AISI 304	AISI 1018	AISI 304
MC: Control/100% OPC	MC-1-18	MC-1-304	MC-2-18	MC-2-304
M10: Mixture/90% OPC, 10% SCBA-SF	M10-1-18	M10-1-304	M10-2-18	M10-2-304
M20: Mixture/80% OPC, 20% SCBA-SF	M20-1-18	M20-1-304	M20-2-18	M20-2-304
M30: Mixture/70% OPC, 30% SCBA-SF	M30-1-18	M30-1-304	M30-2-18	M30-2-304

**Table 7:** The measured half-cell corrosion potential (*E_corr_*) versus a Cu/CuSO_4_ in reinforcement concrete [81,82].

*E_corr_* vs. CSE	Corrosion Condition
*E_corr_* > −200	Low (10% of risk corrosion)
−200 > *E_corr_* > −350	Intermediate corrosion risk
−350 > *E_corr_* > −500	High (<90% of risk corrosion)
*E_corr_* < −500	Severe Corrosion

**Table 8:** Ranges of corrosion current density (*i_corr_*), and the corrosion rate (*v_corr_*) related to corrosion level [87].

*i_corr_* (µA/cm^2^)	*v_corr_* (mm/y)	Corrosion Level
≤0.1	≤0.001	Negligible (Passivity)
0.1–0.5	0.001–0.005	Low Corrosion
0.5–1	0.005–0.010	Moderate Corrosion
>1	>0.010	High Corrosion
